# Response to letter regarding “Clinical features, diagnosis, and survival analysis of dogs with glioma”

**DOI:** 10.1111/jvim.16488

**Published:** 2022-07-27

**Authors:** Roberto José‐López, Rodrigo Gutierrez‐Quintana, Martí Pumarola, Cristian de la Fuente, Sonia Añor, Edgar G. Manzanilla, Anna Suñol, Dolors Pi Castro, Daniel Sánchez‐Masian, Francisco Fernández‐Flores, Katia Marioni‐Henry, Lara A. Matiasek, Kaspar Matiasek, Emanuele Ricci

**Affiliations:** ^1^ Hamilton Specialist Referrals—IVC Evidensia High Wycombe United Kingdom; ^2^ School of Veterinary Medicine, College of Medical, Veterinary and Life Sciences, University of Glasgow Glasgow United Kingdom; ^3^ Department of Animal Medicine and Surgery, Veterinary Faculty Universitat Autònoma de Barcelona Barcelona Spain; ^4^ TEAGASC, The Irish Food and Agriculture Authority Cork Ireland; ^5^ Anicura ARS Veterinaria Barcelona Spain; ^6^ Anicura Arvivet Hospital Veterinari Barcelona Spain; ^7^ Hospital de Referencia Veterios Madrid Spain; ^8^ Uranovet Barcelona Spain; ^9^ Royal (Dick) School of Veterinary Studies and Roslin Institute, University of Edinburgh Edinburgh United Kingdom; ^10^ Frontier Veterinary Specialists Hergolding Germany; ^11^ Centre for Clinical Veterinary Medicine Ludwig‐Maximilians‐Universitaet Muenchen Munich Germany; ^12^ Department of Veterinary Anatomy, Physiology and Pathology University of Liverpool Neston United Kingdom

Dear Editor,

Thank you for the opportunity to respond to the letter from Drs Rohrer Bley, Meier, Beckmann, and Steffen regarding our paper in JVIM titled “Clinical features, diagnosis, and survival analysis of dogs with glioma.” We appreciate their interest in our study especially with regard to the discussion of our findings on treatment and survival in our group of dogs with histologically confirmed glioma.

The aims of our study were to further characterize the clinicopathologic and diagnostic imaging features of a large cohort of dogs with glioma as well as analyzing survival in those which received any type of therapy. We are happy to see that there were no concerns with our observations on the clinicopathologic and imaging features of the cases included. We were able to study survival in 45 of the included dogs, the largest cohort with histopathologic diagnosis of glioma that we are aware of reported to date. Despite limitations, this was of particular relevance as available information on treatment and outcome of dogs with histologically confirmed diagnosis of glioma treated with any modality was anecdotal until our publication. Most importantly, we defined survival in a relatively large group of dogs receiving palliative therapies (ie, corticosteroids, antiepileptic, or analgesic medications as sole therapeutic intervention), which informs future studies evaluating responses to other conventional or experimental therapies. For analysis purposes and due to the limitations imposed by case numbers, we grouped dogs receiving surgery, radiotherapy or chemotherapy in any combination together, similarly to a recent study of histiocytic sarcoma affecting the CNS.^1^ We would have preferred to refer to this group as receiving “specific” treatment modalities; however, we were invited to use “definitive” treatment instead by the reviewers to, although admittedly not ideal, stay in line with the aforementioned publication.^1^ The term “definite treatment” used in our paper did not refer to the efficacy of the chosen treatment modality but to the fact to treat a confirmed neoplasia by a presumed antineoplastic therapy.

Rohrer Bley and colleagues referred to a systematic review on brain tumor treatment in dogs to suggest that radiotherapy might be more effective than surgery at treating intracranial tumors in dogs and that chemotherapy is not an acceptable option for such purpose.^2^ However, that review failed to show a clear difference in outcome between radiotherapy and surgery. Furthermore, most of the studies evaluated in that review lacked histologic diagnosis of the lesion thereby limiting the value of their observations. Similarly, the subsequent studies Rohrer Bley and colleagues referenced to reiterate the superiority of radiotherapy over surgery in the treatment of intracranial gliomas, lacked histologic confirmation for any of the masses treated. Those studies included cases “suspected” of having intracranial glioma based on MRI characteristics; however, other tumor types or intraaxial brain lesions such as cerebrovascular accidents or granulomas can mimic gliomas on MRI.^3^, ^4^ Potential inclusion of these can bias results toward more favorable outcomes. Thus, liberally comparing outcomes and survival after radiotherapy in dogs with unconfirmed glioma with surgery in histologically confirmed cases^5^ is not accurate and could mislead readers.

To avoid that source of bias, we only included dogs with histopathologically confirmed glioma in our analysis. Radiotherapy is routinely offered as a therapeutic option for dogs with suspected glioma in all the institutions contributing to our study, either alone or in combination with surgery for diagnosis and tumor debulking to alleviate clinical signs. Unfortunately, only 2 dogs treated with radiotherapy alone or in combination with surgery met our inclusion criterion. Radiation therapy specifics were not provided due to this and the restrictions to manuscript extension. Dogs receiving chemotherapy in our study did so as their owners declined radiotherapy and surgery but elected this option in addition to palliative therapy either by enrolling in a clinical trial or administering lomustine or temozolomide. Lomustine treatment was associated with longer survival than palliative therapy in dogs with presumptive intracranial glioma in a recent study albeit most of the included cases lacked histopathologic diagnosis,^6^ and temozolomide is a standard‐of‐care for adjuvant and monotherapy of high‐grade gliomas in humans and its use is being actively investigated in dogs.^7^ Finally, dogs in our study that received cytarabine were suspected to have a noninfectious granuloma based on MRI.

Limitations of our survival analysis, mostly related to its retrospective nature, low case numbers and marked variability in treatment modalities, are well stated in the paper to assist with interpretation of results. It was never inferred in the paper that any of the more specific treatment modalities is superior to others, instead, suggestion was made that specific therapies in general provide a survival benefit to dogs with confirmed intracranial gliomas in comparison to palliative treatment.

With regards to the concerns risen about recommendations for euthanasia, presumptive diagnosis of glioma at any of the institutions contributing to our paper was always followed by a thorough discussion with the owners regarding most likely diagnoses, their respective prognosis, and treatment options including the abovementioned specific therapeutic modalities and palliative treatment. Minimally invasive techniques (including stereotactic brain biopsies) to obtain a definitive diagnosis and a maximally informed approach to treatment are also offered to owners at the contributing institutions. Despite the available options, many owners still elected to euthanize in view of the likely poor prognosis and the severity of the clinical status of some dogs, or due to financial constraints. Owner decision to euthanize is a common issue in veterinary medicine and, as stated in the paper, a limitation to analysis of glioma behavior in the light of treatment.

Finally, another aim of our study was to assess the relationship between clinicopathologic features and survival and glioma histologic type and grade, based on the Comparative Brain Tumor Consortium diagnostic classification. Tumor volume determined via MRI was neither associated with nor predictive of outcome in a recent study including 60 dogs with histologically confirmed glioma, thus, it was not included in our analysis.^5^

We failed to identify associations between survival and glioma type or grade. As acknowledged in our discussion, this could be related to the low number of cases (36) receiving any treatment, surviving >1 day after imaging diagnosis, and with MRI available for revision included in our multivariable analysis of prognostic factors affecting survival. Again, marked variability in treatment modalities might have also influenced. By contrast, a subsequently published study including 33 gliomas, all treated with surgical resection and immunotherapy, identified astrocytomas and low‐grade tumors were associated with increased survival.^8^ Whereas the latter study findings result from univariable analysis ours resulted from univariable followed by multivariable Cox proportional hazard modeling. Therefore, differences between both studies' findings might be attributable to methodology of statistical analysis and lack of homogeneity of treatment modalities.

Further evidence based on larger case numbers and multivariable statistical analysis is necessary to confirm any association between glioma type or grade and prognosis in dogs. Thus, we take this opportunity once again to invite researchers to contribute to the creation of a mutually accessible international multicenter database to better enable evidence‐based research in the field of canine glioma.
